# Tension-type headache associated with obstructive sleep apnea: a nationwide population-based study

**DOI:** 10.1186/s10194-015-0517-5

**Published:** 2015-04-21

**Authors:** Yu-Chwen Chiu, Hsiao-Yun Hu, Fei-Peng Lee, Hung-Meng Huang

**Affiliations:** Department of Otolaryngology, Taipei City Hospital, Taipei, Taiwan; Department of Education and Research, Taipei City Hospital, Taipei, Taiwan; Department of Public Health, Institute of Public Health, National Yang Ming University, Taipei, Taiwan; Department of Otolaryngology, School of Medicine, College of Medicine, Taipei Medical University, Taipei, Taiwan; Department of Otolaryngology, Wan Fang Hospital, Taipei Medical University, Taipei, Taiwan

**Keywords:** Polysomnography, Sleep study, Health insurance database, Taiwan, Sleep disturbance, Headache, Hypopnea

## Abstract

**Background:**

There is still controversy regarding the association between primary headaches and obstructive sleep apnea. We explored the relationship between tension-type headache (TTH) and obstructive sleep apnea (OSA) using a large nationwide population-based data set in Taiwan.

**Methods:**

We identified 4759 patients diagnosed with OSA from the Taiwan Longitudinal Health Insurance Database, based on polysomnography, as the OSA group. We then randomly selected 19036 subjects without OSA, matched by sex and age, to serve as the non-OSA group. The multivariate Cox proportional hazards model with matching for age and sex was used to assess the possible associations between TTH and OSA among the patients.

**Results:**

The prevalence of TTH was 10.2% among OSA patients and 7.7% among non-OSA patients (*p* < 0.001). The multivariate Cox proportional hazards model revealed patients with OSA were more likely to have TTH (hazard ratio, 1.18; 95% CI, 1.06–1.31) (*p* = 0.003) than patients in the non-OSA group.

**Conclusion:**

Patients with OSA had a higher likelihood of developing TTH than patients in the non-OSA group. Further studies of physiological patterns between OSA and TTH are needed to confirm the study findings.

## Background

Obstructive sleep apnea (OSA) is by far the most common sleep disturbance, affecting some 24% of men and 9% of women by the time they reach middle age [1]. Repeated episodes of apnea or hypopnea caused by upper airway blockage during sleep characterize OSA. A typical measurement of sleep apnea is the apnea-hypopnea index. This is an average that represents the combined number of episodes of apnea and hypopnea that occur per hour of sleep, which is calculated from the results of overnight polysomnography. The definitive diagnostic test for OSA is polysomnography. Obstructive sleep apnea is receiving increased attention as an important cause of medical morbidity, including excessive daytime sleepiness, metabolic derangement, and cardiovascular disease.

People in the general population frequently experience headache pain, placing a burden on health care and other economic expenditures. Headaches may be intrinsically related to sleep, may cause sleep disturbances, or manifestations of sleep apnea. The International Classification of Headache Disorders (ICHD II) [2] includes two specific diagnoses for sleep-related headaches, “sleep apnea headache” and “hypnic headache”. Sleep apnea headaches present upon awakening with an apnea-hypopnea index ≥ 5, as demonstrated by overnight PSG. Hypnic headaches develop only during sleep and always awaken patients. Nevertheless, sleep disorders may still be associated with other primary headaches, especially tension-type headaches (TTH).

Several studies [3-6] have illustrated positive correlations between TTH and sleep disturbances including sleep apnea. Nevertheless, several studies [7-9] have shown no correlation. In a recent cross-sectional population-based study, Kristiansen et al. [8,9] showed that sleep apnea had little if any influence on the development of TTH. Therefore, the goal of this research was to explore the possible association between TTH and OSA within a nationwide, population-based data set in Taiwan.

## Methods

### Data sources

Taiwan’s National Health Insurance (NHI) program, which started March 1, 1995, provides comprehensive coverage for medical care of people living in Taiwan. This program was built on the concept of mutual assistance, and it depends on the insured paying their premiums according to regulations. At the end of 2012, there were 23 million people (approximately 99% of Taiwan’s population) enrolled in the program. The Bureau of NHI collects data from the NHI program and sends it to the National Health Research Institutes (NHRI) to form the original files of National Health Insurance Research Database (NHIRD). The study used data from the Longitudinal Health Insurance Database 2000 (LHID2000), which was created by the NHRI and contained all the original claims data for one million individuals (approximately 5% of Taiwan’s population) randomly sampled from the 2000 Registry of Beneficiaries of the NHIRD. The NHRI confirmed that there was no significant difference in gender distribution between the patients in the LHID2000 and the original NHIRD.

All information allowing a specific patient to be identified was encrypted. The encryption procedure was consistent between datasets; thus, all claims belonging to each patient were linked. The confidentiality of the data abides by the data regulations of the Bureau of National Health Insurance. The Institutional Review Board of Taipei City Hospital approved this study (IRB no.: TCHIRB-1020715-W).

### Study sample

We first identified all patients with a new diagnosis of OSA (International Classification of Diseases, Ninth Revision, Clinical Modification [ICD-9-CM] codes 327.23, 780.51, 780.53, or 780.57) based on polysomnography (AHI *≥* 5) between January 1, 2004 and December 31, 2010 (n = 5023). We excluded patients younger than 18 years from the analysis (n = 264). For each case, the date of the first diagnosis was assigned as the index date. Finally, the OSA group included 4759 patients.

We selected 19,036 patients without OSA (four for each OSA case) from the remaining subjects of the LHID2000 as the non-OSA group. The patients in the non-OSA group were matched with the case subjects in OSA group by sex, age group (<30, 30–39, 40–49, 50–59, ≥ 60), and year of the index date. None of the selected non-OSA patients had been diagnosed with OSA after 1995, the initiation year of the NHI program.

We also investigated the pre-existing comorbidities of hypertension (ICD-9-CM 401), epilepsy (ICD-9-CM 345), major depression disorders (ICD-9-CM 296.2 and 296.3), and anxiety disorders (ICD-9-CM 300.00, 300.01, 300.02 and 300.09). We selected these comorbidities due to their possible associations with OSA and TTH as documented in earlier studies [10-14].

Cases of TTH were identified based on their corresponding ICD-9-CM codes. We used ICD-9-CM codes 307.81 and 339.1 for TTH. Neurologists diagnosed all headaches. We included patients in this group only when TTH was diagnosed after the OSA index date.

### Statistical analysis

SAS version 9.3 (SAS Institute Inc., Cary, NC, USA) was used to link the data in the LHID2000; Stata 13 (Stata Corporation, College Station, TX, USA) was used to perform the statistical analyses. Demographic information of patients with or without OSA were compared using a *χ*2 test for categorical variables. The threshold for statistical significance was set at two-tailed P value < 0.05. The risk of TTH among OSA patients and OSA-free group between January 1, 2004 and December 31, 2010 was analyzed. The log-log survival curve was used to test the proportional assumption. The log-log plots indicated that the data might be suitable for the Weibull distribution parameters estimation. A sensitivity test of the Weibull model was performed. Cox proportional hazards regression model was used to estimate the adjusted hazard ratio (HR) and 95% confidence intervals (CI) for developing TTH. The analysis included potential correlation factors of TTH such as hypertension, epilepsy, major depression disorder, and anxiety disorder. Finally, HRs were calculated for specific subgroups with adequately adjusted regression models.

## Results

After matching for sex and age, we found that patients with OSA had higher proportions of certain comorbidities than the non-OSA group did (Table 1), including hypertension (38.3% vs. 30.2%, *p* < 0.001), epilepsy (2.5% vs. 1.6%, *p* < 0.001), major depression disorder (5.5% vs. 2.7%, *p* < 0.001), and anxiety disorder (26.7% vs. 15.3%, *p* < 0.001). Table 1 shows the relationships between sociodemographic characteristics and OSA. Patients with OSA were more likely to have monthly incomes of NT$ 40,000 or higher (*p* < 0.001) and live in northern and central Taiwan (*p* < 0.001).

**Table 1:** **Demographic characteristics of patients with obstructive sleep apnea (OSA) and non-OSA-diagnosis patients from 2004 to 2010**

**Variable**	**Matched controls**		**Patients with OSA**		***p*** **-value**
	**(N = 19036)**	**%**	**(N = 4759)**	**%**	
Sex					1.000
Female	5,524	29.0	1,381	29.0	
Male	13,512	71.0	3,378	71.0	
Age					1.000
<30	1,956	10.3	489	10.3	
30-39	3,780	19.9	945	19.9	
40-49	4,720	24.8	1,180	24.8	
50-59	4,768	25.0	1,192	25.0	
≥60	3,812	20.0	953	20.0	
Monthly Income, NT$					<0.001
<20,000	2,285	12.0	515	10.8	
20,000-39,999	6,599	34.7	1,461	30.7	
≥40,000	4,447	23.4	1,588	33.4	
Union/association member	2,647	13.9	455	9.6	
Local government enrollee	3,058	16.1	740	15.5	
Region					<0.001
Northern	8,529	44.8	2,476	52.0	
Central	4,131	21.7	1,434	30.1	
Southern	5,943	31.2	811	17.0	
Eastern	433	2.3	38	0.8	
Comorbidity					
Hypertension	5,755	30.2	1,823	38.3	<0.001
Epilepsy	297	1.6	121	2.5	<0.001
Major depression disorder	520	2.7	264	5.5	<0.001
Anxiety disorder	2,911	15.3	1,270	26.7	<0.001
Tension type headache					<0.001
No	17,567	92.3	4,275	89.8	
Yes	1,469	7.7	484	10.2	
Total	19,036		4,759		

The prevalence of TTH was 10.2% among OSA patients, and was higher than in non-OSA patients (7.7%, *p* < 0.001).

Table 2 shows the incidence rates (IR) in each group over the 6-year follow-up period. The incidence rate of TTH was higher in OSA patients (37 per 1000 person-years) than in non-OSA patients (28 per 1000 person-years).

**Table 2:** **The incidences of tension-type headache in each group from 2004 to 2010**

**Variables**	**Tension-type headache**		
	**Person-Years**	**TTH (N)**	**IR (‰/yr)**
Total	64,864	1,953	30
Sex			
Female	18,102	810	45
Male	46,762	1,143	24
Age			
<30	6,938	104	15
30-39	13,122	316	24
40-49	16,460	464	28
50-59	15,996	579	36
≥60	12,348	490	40
Monthly Income			
<20,000	9,122	217	24
20,000-39,999	20,610	628	30
≥40,000	16,365	384	23
Union/association member	8,564	363	42
Local government enrollee	10,202	361	35
Region			
Northern	30,023	749	25
Central	14,865	448	30
Southern	18,709	719	38
Eastern	1,266	37	29
Comorbidy			
Hypertension	21,434	882	41
Epilepsy	1,153	34	29
Major drpression disorder	2,226	177	80
Anxiety disorder	11,579	769	66
Obstructive sleep apnea			
No	51,824	1,469	28
Yes	13,040	484	37

*p*-value < 0.001 in all results.

IR: incidence rate.

We used multivariate Cox proportional hazards regression model for predicting the chances of developing TTH (Table 3). After adjustment for sex, age, sociodemographic factors, and comorbidities, patients with OSA were more likely to have TTH (HR = 1.18, 95% CI 1.06–1.31, *p* = 0.003) than patients in the non-OSA group. Patients with hypertension tended to have TTH (HR = 1.28, 95% CI 1.16–1.42, *p* < 0.001).

**Table 3:** **Multivariate Cox regression model for predictor of tension-type headache development**

**Variable**	**Tension-type headache**		
	**HR**	**95% CI**	***p*** **-value**
Sex			
Female	1.00		
Male	0.61	0.56-0.67	<0.001
Age			
<30	1.00		
30-39	1.56	1.25-1.94	<0.001
40-49	1.58	1.27-1.96	<0.001
50-59	1.76	1.42-2.18	<0.001
≥60	1.65	1.31-2.06	<0.001
Monthly Income			
<20,000	1.00		
20,000-39,999	1.34	1.14-1.56	<0.001
≥40,000	1.09	0.92-1.30	0.298
Union/association member	1.51	1.27-1.80	<0.001
Local government enrollee	1.35	1.14-1.60	<0.001
Region			
Northern	1.00		
Central	1.09	0.96-1.23	0.173
Southern	1.50	1.35-1.67	<0.001
Eastern	1.06	0.76-1.48	0.720
Comorbidy			
Hypertension	1.28	1.16-1.42	<0.001
Epilepsy	0.79	0.56-1.11	0.170
Major drpression disorder	1.73	1.47-2.03	<0.001
Anxiety disorder	2.30	2.09-2.54	<0.001
Obstructive sleep apnea			
No	1.00		
Yes	1.18	1.06-1.31	0.003

Among affective disorders, patients with major depression disorder were more likely to have TTH (HR = 1.73, 95% CI 1.47–2.03, *p* < 0.001). Patients with anxiety disorder also had greater chances of developing TTH (HR = 2.30, 95% CI 2.09–2.54, *p* < 0.001).

We found that men had a lower chance of having TTH (HR = 0.61, 95% CI 0.56–0.67, *p* < 0.001) than did women. TTH occurred more often among older age adults (age 50–59 years, HR = 1.76, 95% CI 1.42–2.18, *p* < 0.001).

Regarding economic condition and geographic location, we found that the chances of developing TTH increased when monthly income was less than NT$ 20,000 and patients resided outside of northern Taiwan.

Table 4 shows the subgroup analysis of the variables. Regardless of gender, patients with OSA were more likely to have TTH than were non-OSA patients. Patients with OSA 50–59 years of age with monthly income of NT$ 40,000 or more had a statistically significant association with TTH. All OSA patients, even those without the selected comorbidities, showed a statistically significant association with TTH compared to non-OSA patients.

**Table 4:** **Risk comparison of tension-type headache between OSA and non-OSA participants**

**Variable**	**N**	**Tension-type headache**	
		**HR**	**95% CI**
Sex			
Female	6,905	1.17	1.00-1.39
Male	16,890	1.17	1.02-1.35
Age			
<30	2,445	1.27	0.80-2.00
30-39	4,725	1.03	0.78-1.35
40-49	5,900	1.40	1.13-1.73
50-59	5,960	1.29	1.06-1.56
≥60	4,765	0.93	0.74-1.17
Monthly Income			
<20,000	2,800	1.05	0.75-1.47
20,000-39,999	8,060	1.16	0.96-1.41
≥40,000	6,035	1.37	1.11-1.70
Union/association member	3,102	0.88	0.65-1.18
Local government enrollee	3,798	1.22	0.96-1.54
Region			
Northern	11,005	1.22	1.04-1.43
Central	5,565	1.25	1.02-1.53
Southern	6,754	0.99	0.80-1.23
Eastern	471	2.47	0.90-6.81
Comorbidity			
Hypertension			
No	16,217	1.22	1.06-1.42
Yes	7,578	1.08	0.92-1.26
Epilepsy			
No	23,377	1.19	1.07-1.32
Yes	418	0.75	0.31-1.82
Major depression disorder			
No	23,011	1.18	1.06-1.32
Yes	784	1.18	0.86-1.61
Anxiety disorder			
No	19,614	1.25	1.08-1.45
Yes	4,181	1.10	0.94-1.28

Each factor was adjusted for all other factors.

## Discussion

According to our results, obstructive sleep apnea is associated with gender, age, sociodemographic characteristics, metabolic consequences, and affective disorders (Table 1). TTH is the most common primary headache. Nonetheless, there has been much less study of TTH compared with migraine. It is likely that patients suffer less with TTH than other chronic headaches. A recent study showed that headache patients who do not seek medical assistance are more likely to have TTH [15]. TTH is associated with certain mental illnesses, especially anxiety and major depression disorder [16]. We extracted possible overlapping variables from the database to make clear their direct or indirect effects on migraine and TTH.

In accordance with our findings, one cross-sectional population based study with 297 participants enrolled from Norway used a modified short form of the Karolinska Sleep Questionnaire and Epworth Sleepiness Scale. They found severe sleep disturbance was three times more likely for individuals with TTH [6]. Authors of another retrospective analysis reported that among 82 chronic headache patients with migraine or TTH or both, 52 patients (63%) also had OSA. The headache improvement rate was up to 49% after receiving either standard medical therapy or continuous positive airway pressure therapy [17].

The main finding of our study showed a positive relationship between OSA and TTH. Regardless of gender, patients with OSA were more likely to have TTH after adjustment for age, residential region, monthly income, and possible comorbidities. In Table 4, patients with OSA showed stronger positive associations with TTH in most comorbidity-free groups than in comorbidity groups. We assumed patients with the selected comorbidities had poorer health-related quality of life and did not undergo OSA testing, which is not considered an emergency, but needs to be confirmed by a time-consuming polysomnography study.

Lack of sleep itself is related to headaches. Common sleep disorders include OSA, central sleep apnea, insomnia, periodic limb movement disorder [18], and bruxism [19]. The mechanisms by which headaches are associated with specific sleep disturbances are complex and manifold. In cluster headaches, oxygen desaturation below 89% during sleep preceded 8 out of 14 attacks of cluster headache among 10 patients [20], giving positive support for the relationship of cluster headache and OSA.

Serotonin and several other neurotransmitters influence TTH. Pain thresholds elicited in patients with chronic, painful pathologies are linked to serum serotonin levels [21]. Additionally, many TTH patients show changes in serotonin levels [22-26]. Interestingly, serotonin dysfunction may also play a role in OSA [27,28]. Serotonin delivery is reduced to upper airway dilator motor neurons during sleep, and this contributes to sleep-related reductions in dilator muscle activity and thus, upper airway obstruction [27]. Fluctuation of serotonin concentrations could be a primary or secondary factor common to different mechanisms. Such a hypothesis provides a possible explanation for the manifestation of OSA and TTH. Furthermore, a growing number of studies show that sleep disturbance increases pain sensitivity and exacerbates chronic pain, including fibromyalgia, rheumatoid arthritis, and headache [29-31]. Smith et al. [32] suggested that sleep fragmentation impairs endogenous pain inhibitory function and increases spontaneous pain in middle-aged healthy women. Lentz et al. [33] found that slow wave sleep disruption without reducing total sleep was also associated with decreased pain thresholds in middle-aged women. Khalid et al. [34] demonstrated that patients with severe OSA were hyperalgesic, and their pain sensitivity improved with continuous positive airway pressure treatment, and the improvement disappeared immediately with discontinuation of continuous positive airway pressure therapy. We suggest patients with refractory TTH undergo evaluation for OSA. Treating OSA might not only improve headaches, but also decrease the risk of related comorbidities.

### Clinical importance and limitations

In this study, we utilized a nationwide population database to investigate the epidemiologic associations between OSA and TTH. One of the key strengths of the study, in addition to a large sample size, was selecting patients with an OSA diagnosis confirmed by polysomnography. We believe this selection criterion is more precise and strict than patient recall in response to a questionnaire. Nonetheless, this could also lead to underestimation of the enrolled OSA patients in the total population. The National Health Insurance Administration in Taiwan samples the medical charts routinely and makes detailed assessments for accuracy of medical coding. The reliability and validity of the NHI research database for epidemiologic investigation has been reported [35].

A key limitation of the study is the relatively low prevalence of OSA and headache. The prevalence of TTH in the general population varies from 30% to 78% (2) because of differences in research study design. A diagnosis of TTH may be overlooked or delayed because symptoms can be mild or infrequent. In addition, some symptoms can mimic TTH of other conditions, such as acute or chronic paranasal sinusitis, transient ischemic attack, meningitis, chronic subdural hemorrhage, and brain tumor. TTH diagnoses of patients enrolled in our study were made by neurologists, which means we may have reliable accuracy of the clinical diagnoses in our study. Similarly, according to epidemiological studies, the prevalence rates of OSA in the general population vary from 3% to 7% [36]. It was about 0.48% (4759/1000000) in our study due to the strict selection of polysomnography-confirmed cases only. A disadvantage of our study is that we could not have face-to-face interviews with patients. Thus, we can only set higher limitations on case selection to lower inaccuracy from such a large study sample. Even if the prevalence were underestimated, we still would have convincing findings.

Another limitation of the study is that the degree of OSA and the pattern of headache attack could not be determined from the database. Thus, we could not determine whether the severity of OSA influenced TTH frequency, duration, and pain characteristics. We could not differentiate TTH from episodic and chronic type headaches. Additionally, some possibly relevant confounding factors, such as body mass index, smoking, and alcohol consumption were not included in the data set. Therefore, they were not analyzed.

In our findings, the incidence rates (Table 2) over the 6-year follow-up period (from 2004 to 2010, 30 to 78 per 1,000 person-years) were higher than some earlier studies (13). Not being able to completely exclude recurrences should be taken into account, especially in older age group. The substantially increasing trend in the incidence of frequent headache in past 10 years was also found in other studies [37]. Additional researches on the occurrence and causes of increasing headache incidences are needed.

## Conclusions

Our results support the notion that patients with OSA are more likely to develop TTH. The mechanism underlying this association is not clear but includes changes in serotonin levels. Further studies of physiological patterns of OSA and TTH are needed to confirm the study findings and to enrich our understanding of their interactions.

## Abbreviations

TTH: Tension-type headache
OSA: Obstructive sleep apnea
NHRI: National health research institutes
NHIRD: National health insurance research database
LHID2000: Longitudinal health insurance database 2000
ICD-9-CM: International classification of diseases, ninth revision, clinical modification
HR: Hazard ratio
CI: Confidence interval
